# Self - evaluation of social adaptation in patients with schizophrenia and metabolic syndrome

**DOI:** 10.1192/j.eurpsy.2021.435

**Published:** 2021-08-13

**Authors:** V. Khamina, E. Kornetova, A. Kornetov, A. Goncharova, A. Boiko

**Affiliations:** 1 Mental Health Research Institute, Tomsk National Research Medical Center of Russian Academy of Sciences, Tomsk, Russian Federation; 2 Department Of Fundamental Psychology And Behavioral Medicine, Siberian State Medical University, Tomsk, Russian Federation

**Keywords:** social adaptation, schizophrénia, Metabolic syndrome

## Abstract

**Introduction:**

Metabolic syndrome (MS) is an often co-occurring condition that occurs during antipsychotic therapy and impairs social functioning

**Objectives:**

We tried to conduct a self - evaluation of social adaptation in patients with schizophrenia and MS

**Methods:**

We examined 150 patients with schizophrenia receiving antipsychotic therapy at the clinics of Mental Health Research Institute. The study was supported by a grant from the Russian Science Foundation 18-15-00011. The IDF criteria were used to diagnose metabolic syndrome. We used «The social adaptation self - evaluation scale» (SASS).

**Results:**

63 patients (42%) had MS and 87 patients (58%) did not. In the subgroup of patients with MS, 59 people (93.65%) had disabilities or were unemployed, in the group without MS - 82 (94.26%) patients. There were no statistically significant differences between the groups (p ≥ 0.05). In the patients with schizophrenia and concomitant MS, the median SSAS scores was 35 [29; 39], which corresponds mainly to a high level of self - evaluation of social adaptation. At the same time, in patients with schizophrenia and without MS, on the contrary, the self - evaluation of social adaptation was 30 [23; 38] points (p = 0.03914). Perhaps this is due to the great attention from relatives and doctors of general somatic practice and the primary medical network in connection with the risk of developing severe somatic pathology.

**Conclusions:**

Patients with MS can give a higher assessment of social adaptation, despite a objectively low social status.

**Disclosure:**

No significant relationships.

